# Evaluation of PTV margin in CBCT-based online adaptive radiation therapy for gastric mucosa-associated lymphoid tissue lymphoma

**DOI:** 10.1093/jrr/rrae052

**Published:** 2024-06-27

**Authors:** Taka-aki Hirose, Masanori Takaki, Yusuke Shibayama, Jun-ichi Fukunaga, Toyoyuki Kato, Tadamasa Yoshitake, Kousei Ishigami

**Affiliations:** Division of Radiology, Department of Medical Technology, Kyushu University Hospital, 3-1-1 Maidashi, Higashi-Ku, Fukuoka 812-8582, Japan; Department of Clinical Radiology, Graduate School of Medical Sciences, Kyushu University, 3-1-1 Maidashi, Higashi-Ku, Fukuoka 812-8582, Japan; Division of Radiology, Department of Medical Technology, Kyushu University Hospital, 3-1-1 Maidashi, Higashi-Ku, Fukuoka 812-8582, Japan; Division of Radiology, Department of Medical Technology, Kyushu University Hospital, 3-1-1 Maidashi, Higashi-Ku, Fukuoka 812-8582, Japan; Division of Radiology, Department of Medical Technology, Kyushu University Hospital, 3-1-1 Maidashi, Higashi-Ku, Fukuoka 812-8582, Japan; Department of Clinical Radiology, Graduate School of Medical Sciences, Kyushu University, 3-1-1 Maidashi, Higashi-Ku, Fukuoka 812-8582, Japan; Department of Clinical Radiology, Graduate School of Medical Sciences, Kyushu University, 3-1-1 Maidashi, Higashi-Ku, Fukuoka 812-8582, Japan

**Keywords:** gastric mucosa-associated lymphoid tissue, planning target volume margin, online adaptive radiotherapy, cone-beam computed tomography, intrafractional motion

## Abstract

The aim of this study was to investigate planning target volume (PTV) margin in online adaptive radiation therapy (oART) for gastric mucosa-associated lymphoid tissue (MALT) lymphomas. Four consecutive patients with gastric MALT lymphoma ﻿who received oART (30 Gy in 15 fractions) on the oART system were included in this study. One hundred and twenty cone-beam computed tomography (CBCT) scans acquired pre- and post-treatment of 60 fractions for all patients were used to evaluate intra- and interfractional motions. Patients were instructed on breath-holding at exhalation during image acquisition. To assess the intrafraction gastric motion, different PTVs were created by isotropically extending the CTV contoured on a pre-CBCT image (CTV_pre_) at1 mm intervals. Intrafraction motion was defined as the amount of expansion covering the contoured CTV on post-CBCT images (CTV_post_). Interfractional motion was defined as the amount of reference CTV expansion that could cover each CTV_pre_, as well as the evaluation of the intrafractional motion. PTV margins were estimated from the cumulative proportion of fraction covering the intra- and interfractional motions. The extent of expansion covering the CTVs in 90% of fractions was adopted as the PTV margin. The PTV margin for intrafractional gastric motion using the oART system with breath-holding was 14 mm. In contrast, the PTV margin for interfractional gastric organ motion without the oART system was 25 mm. These results indicated that the oART system can reduce the PTV margin by >10 mm. Our results could be valuable data for oART cases.

## INTRODUCTION

Most low-grade gastric mucosa-associated lymphoid tissue (MALT) lymphomas respond well to the eradication of *Helicobacter pylori* [1]. However, for gastric MALT lymphomas that persist or recur after *H. pylori* eradication, radiotherapy is one of the most effective treatments and is also indicated in rare manifestations of low-grade early stage MALT lymphoma without *H. pylori* infection [2]. The clinical target volume (CTV) for gastric MALT lymphoma is defined as the entire stomach, whereas the planning target volume (PTV) is defined as the CTV with an additional margin that considers variations in stomach volume, respiratory movement and patient setup error [2]. Excellent local recurrence-free survival and distant recurrence-free survival rates have been reported with moderate-dose radiotherapy, and long-term outcomes are expected [3, 4]. Therefore, modern radiation techniques must be used to deliver a uniform dose to the entire stomach and perigastric lymph nodes while sparing the surrounding organs at risk (OAR), such as the kidneys and liver [5, 6]. However, the stomach is known to have a large interfractional deformation and organ motion owing to stomach filling and respiratory movements [7]. To overcome these issues, online adaptive radiation therapy (oART) may be an effective approach that considers large daily shape and size variations [8]. Liu *et al*. reported that oART could contribute to precise dose delivery to the target and reduce radiation-related toxicities for the interfractional deformation of abdominal organs [9]. Although oART can correct interfractional organ motion by replanning based on the daily anatomy, intrafractional organ motion increases with extended treatment time and must be considered when determining the CTV-to-PTV margin (hereafter PTV margin) determination. While some studies ﻿have evaluated the intrafractional gastric motion caused by respiratory movements using fluoroscopic examinations, repeated CT scans, and ﻿four-dimensional cone-beam computed tomography (4D CBCT) [10–12], the time intervals used for the evaluation of the intrafractional motion in these reports are different from the oART imaging scenario, where long treatment sessions are required to create adapted plans. ﻿To the best of our knowledge, no studies have investigated the intrafractional organ motion in oART for gastric MALT lymphomas. In this study, we evaluated the PTV margins required for intrafractional gastric motion using pre- and post-treatment CBCT images acquired during oART for gastric MALT lymphomas.

## MATERIALS AND METHODS

### Patient characteristics and setup

Four ﻿consecutive patients with gastric MALT lymphoma ﻿who received oART (30 Gy in 15 fractions) with 9 or 12 equidistant field intensity-modulated radiotherapy plans created on the Ethos v2.1 system (Varian Medical Systems Inc., Palo Alto, USA) at our institution between September and December 2023 were included in this study. This study was approved by our Institutional Review Board (approval number 22077-00). All patients were immobilized with a thermoplastic mask and whole-body vacuum bag (ESF-19D, Engineering system, Matsumoto, Japan) to reduce interfractional and intrafractional body movements. Patients were instructed not to eat or drink anything for 5 hours before treatment ﻿to minimize stomach volume. In addition, the patients were administered scopolamine butylbromide (Buscopan®) before treatment for each fraction to reduce gastric peristalsis.

### Image acquisition and target delineation

In the Ethos system, an online adaptive plan is created based on the patient’s anatomy in the CBCT images acquired before treatment for each fraction. Additional CBCT images were acquired immediately after treatment to evaluate intrafractional motion during the oART sessions. One hundred and twenty CBCT scans with a tube voltage of 125 kVp, in-plane pixel size of 0.96 mm and slice thickness of 2.0 mm were acquired pre- and post-treatment of 60 fractions for all patients, which were defined as pre-CBCT and post-CBCT, respectively. Patients were instructed on breath-holding at exhalation during image acquisition and beam delivery, and reproducibility of breath-holding was controlled with a gated window of ±2 mm using a marker on the abdomen.

The CTV was defined as the entire stomach from the gastroesophageal junction to the duodenal bulb. The CTV contours on pre-CBCT images created during oART in the Ethos system were retrospectively reviewed and corrected by a radiation oncologist using a commercially available radiation treatment planning system (Eclipse version 16.1; Varian Medical Systems Inc., Palo Alto, CA, USA) to reduce delineation variability. The CTV contours on the post-CBCT images for each fraction were also determined retrospectively by the same radiation oncologist.

### Intrafractional gastric motion

**Fig. 1:**
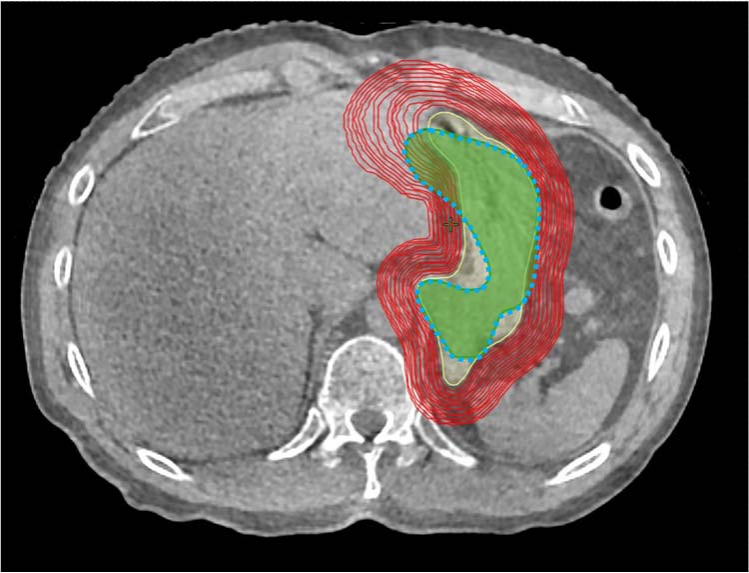
Transverse plane visualization of CTV on pre-treatment CBCT images, different planning target volume margins, and CTV contour on post-treatment CBCT (dashed line).

To assess the intrafractional gastric motion, pre- and post-CBCT images were registered using bone anatomy. PTVs were then created by isotropically extending the CTV contoured on pre-CBCT images (CTV_pre_) at 1 mm intervals (Fig. 1) [13]. Intrafractional motion was defined as the amount of expansion covering the contoured CTV on post-CBCT images (CTV_post_). Furthermore, the oART fraction time from pre- to post-CBCT acquisition was analysed. Volume changes and center of mass (COM) shifts between the CTV_pre_ and CTV_post_ were evaluated. COM shifts were measured in the lateral, vertical and longitudinal directions, and three-dimensional (3D) motion was calculated from the root mean square of each direction.

### Interfractional gastric motion

Interfractional gastric motion was evaluated using CTV_pre_ to compare the effects of intra- and interfractional motions on the required PTV margins with and without the oART system. A reference CTV was used to evaluate the interfractional motion to minimize the influence of stomach volume and respiratory movement variability, which was determined by selecting the average CTV_pre_, excluding CTVs for the upper and lower 30% of the CTV volume and COM shift for each patient. The CTV_pre_ of the remaining 14 fractions excluding the reference CTV for each patient were used for interfractional motion analysis. Interfractional motion was defined as the amount of reference CTV expansion that could cover each CTV_pre_, as well as the evaluation of the intrafractional motion.

### PTV margin estimation for intra- and interfractional motion

The PTV margins were estimated from the cumulative proportion of the fraction covering the intra- and interfractional motions. The extent of expansion covering the CTVs in 90% of fractions was adopted as the PTV margin, in accordance with previous studies that covering 90% of population [13, 14]. All statistical analyses were performed using JMP Pro 16 (SAS Institute Inc., Cary, NC). Statistical significance was set a priori at *P* < 0.05.

## RESULTS

**Fig. 2:**
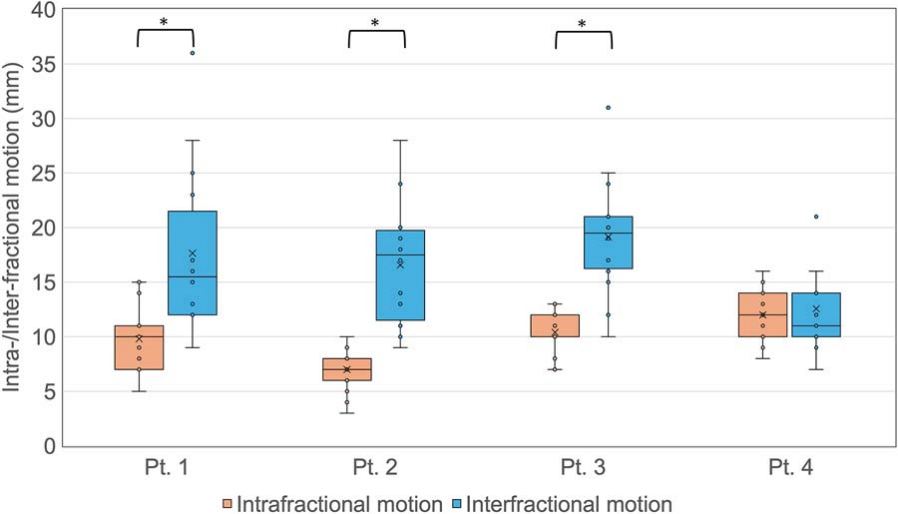
Box plots of intra- and interfractional motion for each patient. The intrafraction motion was defined as the amount of expansion of CTV contoured on pre-treatment CBCT images (CTV_pre_) covering the contoured CTV on post-treatment CBCT images (CTV_post_). The interfractional motion was defined as the amount of reference CTV expansion that could cover each CTV_pre_. (^*^  *P* < 0.05; Mann–Whitney U tests).

Figure 2 illustrates the box plot of the intra- and interfractional motions for each patient. The average intra- and interfractional motion for all fractions were 9.8 ± 2.9 and 16.5 ± 6.2 mm, respectively. The intrafractional motion was significantly smaller than the interfractional motion in patients 1, 2 and 3 (*P* < 0.05; Mann–Whitney U test).

**Fig. 3:**
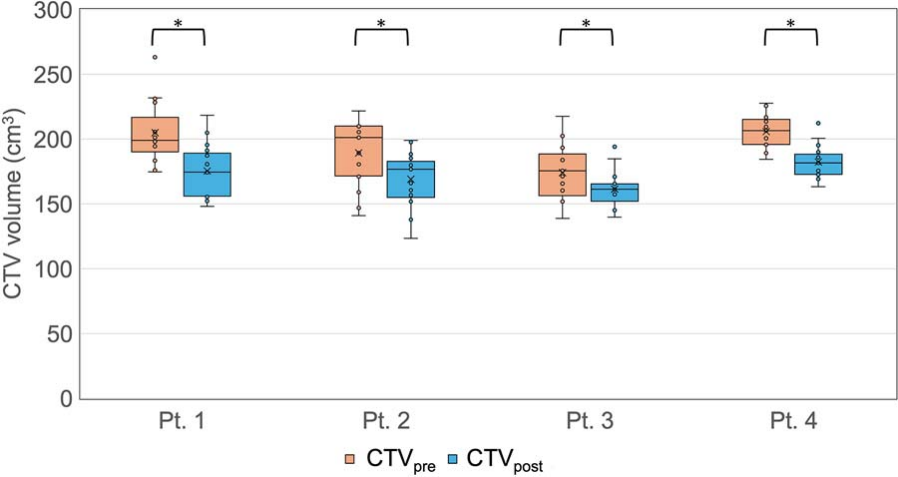
Box plots of CTV volume contoured on pre- and post-treatment CBCT images (CTV_pre_ and CTV_post_) for each patient. (^*^  *P* < 0.05; Wilcoxon signed rank tests).

**Fig. 4:**
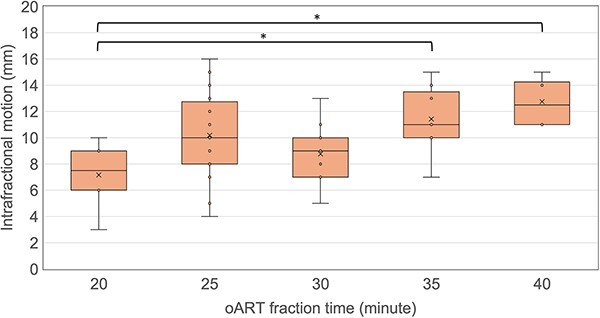
Box plots of intrafractional motion stratified by online adaptive radiation therapy (oART) fraction time. (^*^  *P* < 0.05; Tukey’s tests).

Table 1 shows the mean and standard deviation (SD) of the COM shift between CTV_pre_ and CTV_post_ caused by intrafractional motion. The shift in the longitudinal direction was the largest and the average 3D motion was 5.8 ± 3.6 mm. Figure 3 illustrates a box plot of the CTV_pre_ and CTV_post_ volumes for each patient. The average CTV_pre_ and CTV_post_ volumes for all fractions were 193.5 ± 25.0 and 171.9 ± 19.3 cm^3^, respectively. The CTV volumes of all patients decreased significantly during the oART sessions (*P* < 0.05; Wilcoxon signed-rank test). The average oART fraction time was 30.2 ± 5.6 minutes. Figure 4 shows a box plot of the intrafractional motion stratified by the oART fraction time. The intrafractional motion in ≥35 minutes groups was significantly greater than in <25 minutes group (*P* < 0.05; Tukey’s tests).

**Table 1:** Mean and standard deviation (SD) of center of mass (COM) shift between CTV contoured on pre- and post-treatment CBCT images (CTV_pre_ and CTV_post_) caused by intrafractional motion during online adaptive radiation therapy (oART)

	Lateral	Vertical	Longitudinal	3D motion
COM shift (mm) (Mean $\pm$ SD)	0.4 ± 2.3	0.4 ± 4.3	−1.5 ± 4.6	5.8 ± 3.6

**Fig. 5:**
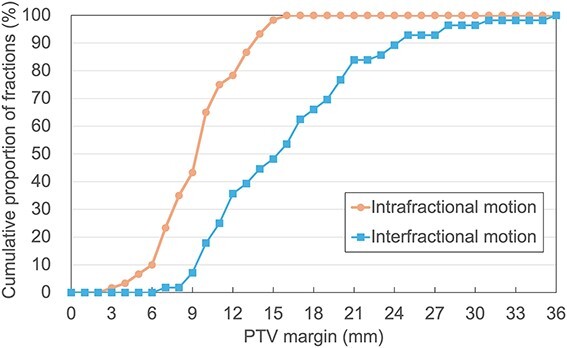
Cumulative proportion of fraction covering intra- and interfractional motion by different PTV margin sizes.

Figure 5 shows the cumulative proportion of the fraction covering the intra- and interfractional motions. ﻿The PTV margins covering the CTV in 90% of the fractions for intra- and interfractional motion were 14 and 25 mm, respectively.

## DISCUSSION

This study evaluated the PTV margin required for intrafractional gastric motion using pre- and post-treatment CBCT images acquired during oART using the Ethos system for gastric MALT lymphomas. The PTV margin for intrafractional gastric motion using the oART system with breath-holding was 14 mm with oART system. In contrast, the PTV margin for interfractional gastric organ motion without the oART system was 25 mm. These results indicate that the oART system can reduce the PTV margin by more than 10 mm.

Regarding intrafractional gastric motion, our results revealed that a PTV margin of 14 mm was sufficient to cover the intrafractional motion for gastric MALT lymphoma during the oART sessions, which took ~30 minutes. Although some studies have investigated intrafractional gastric motion [10–12], most studies have evaluated respiratory movements as intrafractional motion. Therefore, they did not consider organ motion or deformation before and after treatment. Bleeker *et al.* [7] evaluated the intrafractional motion of 65 fractions using end-exhale phase CBCT images before and after treatment. The ﻿displacement of stomach COM ranged from 0.98 to 8.2 mm ﻿(median: 3.6 mm) in median treatment duration ﻿between the pre- and post-CBCT acquisition of 13 minutes. The average COM displacement in the current study was 5.8 mm, which is larger than that in their study (Table 1). It is thought that the extension of the treatment time with oART leads to an increase in intrafractional motion owing to organ deformation and volume changes. When the oART session time was long, the intrafractional motion tended to increase in our results as well (Fig. 4). To the best of our knowledge, no studies have focused on intrafractional motion in oART for gastric MALT lymphomas. Our results could be valuable data for oART cases.

However, the PTV margins of 10 mm or more were larger than those of the treatments at other sites. It is possible to ﻿acquire verification CBCT images and apply couch shifts immediately before the beam-on in the oART workflow of the Ethos system [13]. Although intrafractional motion was assessed by matching based on the bony anatomy in this study, the PTV margin for intrafractional motion may be further reduced by correction based on stomach COM using verification CBCT images for each fraction.

We also evaluated the interfractional motion to compare the effects of oART on the PTV margins. Consequently, a margin of 25 mm was required to cover the CTV interfractional motion. There are several reports regarding interfractional motion. Johnson *et al*. investigated a uniform margin of 22 mm that covered 95% of the stomach contour over the treatment course using daily megavoltage CT with bone matching in gastric lymphoma radiotherapy in three patients [15]. Watanabe *et al.* [11] reported that the PTV margins for the interfractional motion of the center of the stomach obtained from repeated CT scans of six patients were ﻿15.9, 31.0 and 29.6 mm in the longitudinal, ﻿lateral and vertical directions, respectively. Regarding interfractional gastric motion, the results of this study are consistent with those of previous studies.

Our study has some limitations. First, the study included a small number of patients. However, as the results of the interfractional motion were comparable to those of other studies, it can be deduced that the results of the intrafractional motion were also acceptable. Moreover, we could not observe the target position during the beam delivery. It can be affected by respiratory movements. Therefore, the target position, including the reproducibility of breath-holding during image acquisition, was used for evaluation in this study.

## CONCLUSION

In conclusion, the PTV margin for intrafractional motion with breath holding during oART using the Ethos system for gastric MALT lymphomas was 14 mm. The oART reduced the PTV margin by >10 mm. We believe that our results provide useful data for the era of oART.

## PRESENTATION AT A CONFERENCE

None declared.

## CLINICAL TRIAL REGISTRATION NUMBER IF REQUIRED

No.
